# A prospective study of sleep status, anxiety, and depression levels of college students at a university in Shandong Province, China

**DOI:** 10.3389/fpsyg.2024.1361632

**Published:** 2024-04-22

**Authors:** Chengshuai Zhang, Ling Zhao, Tingting Dong, Ji Zhao, Cui Gao, Feng Zhao

**Affiliations:** Shengli Oilfield Central Hospital, Dongying, China

**Keywords:** sleep status, anxiety, depression, students, gender

## Abstract

**Objective:**

To investigate the changes in sleep conditions, anxiety, and depression levels among college students before and after entering the university.

**Methods:**

Utilizing a random sampling method, 692 new students from a college in Shandong province were selected in September 2019, and relevant indices were statistically analyzed in September 2021 following a comprehensive follow-up. Sleep status, anxiety, and depression levels were assessed using the Pittsburgh Sleep Quality Index (PSQI), Patient Health Questionnaire-9 (PHQ-9), and Generalized Anxiety Disorder-7 (GAD-7), respectively.

**Results:**

Gender, passive smoking, exercise, intake of fruits, and intake of seafood were identified as significant influencing factors on college students’ sleep status, anxiety, and depression levels (*p* < 0.05). A substantial difference was observed in the sleep quality of college students between the early enrollment stage and the follow-up stage (*p* < 0.05). Moreover, a significant positive correlation was found between PSQI scores and the levels of anxiety and depression (*p* < 0.05), cumulatively explaining approximately 10% of the variance in anxiety and depression levels.

**Conclusion:**

The sleep quality of college students exhibited significant improvement after enrollment compared to the early enrollment period. Engaging in appropriate exercise and consuming fruits and seafood demonstrated a positive impact on sleep conditions, anxiety, and depression levels. These findings underscore the importance of fostering healthy lifestyle habits for promoting overall well-being among college students.

## 1 Introduction

College students serve as the linchpin for the ongoing progression of society, and the college years represent a pivotal stage between adolescence and adulthood (Cuijpers et al., 2019). Throughout this period, various external factors, such as shifting social roles, escalating pressures, and diminishing social support, coupled with the incomplete construction of self-identity, contribute to the susceptibility of students. In the context of rapidly evolving self-awareness yet a lack of a well-defined personal value system, they become highly vulnerable to the impact of environmental or external influences, culminating in the manifestation of anxiety, depression, and other psychological challenges (Liu et al., 2019; Meckamalil et al., 2022). Numerous studies in recent years have underscored a notable increase in the prevalence of anxiety and depression symptoms among Chinese college students, surpassing those observed in the general population and emerging as a significant risk factor for suicide (Fu et al., 2020; Gao et al., 2020). The World Health Organization’s global health burden survey reveals a steady rise in anxiety and depression worldwide, with a striking surge of 28 and 26% in severe depression and anxiety, respectively, in 2020. This surge is particularly pronounced among individuals aged 20–24 years (Santomauro et al., 2021). These findings indicate a prevailing trend of anxiety and depression within the college student demographic, with an alarming shift toward a younger age group. Research has consistently demonstrated that sleep status significantly influences anxiety and depression in college students (Nyer et al., 2013). Various studies have highlighted suboptimal sleep quality among university students globally, including an Australian study reporting poor sleep quality in 62% of university students (Di Benedetto et al., 2020), Additionally, Serbian scholars investigating medical undergraduates in Pabol found room for improvement in sleep quality within this cohort (Chouhan et al., 2023). Despite these findings, limited research has simultaneously examined sleep quality, anxiety, and depression among university students. Consequently, this study delved into the sleep patterns, anxiety levels, and depression tendencies of college students enrolled in a university in Shandong Province at the commencement of their studies in 2019. The investigation included a follow-up in 2021 to track changes over time, aiming to furnish empirical evidence for more effective interventions and holistic development strategies.

## 2 Methods

### 2.1 Sample size determination

The sample size for this study was determined based on the following considerations. Firstly, to detect changes in sleep conditions, anxiety, and depression levels among college students before and after entering university, a sample size calculation was performed using standard statistical methods. The required sample size was estimated to achieve adequate statistical power to detect meaningful differences in the outcomes of interest. Additionally, considering potential attrition rates and the need for a comprehensive follow-up, a larger sample size was deemed necessary to ensure the robustness and generalizability of the findings.

### 2.2 Sampling procedure

A random sampling method was employed to select participants from a college in Shandong province. In September 2019, 692 new students were randomly selected from the college’s enrollment records to participate in the study. The selection process ensured representation from diverse demographic backgrounds and academic disciplines within the student population. Participants were recruited through invitations sent via email or posted on notice boards, with voluntary participation encouraged.

### 2.3 Comprehensive follow-up

Following the initial data collection in September 2019, participants were followed up in September 2021 to assess changes in sleep conditions, anxiety, and depression levels over time. Comprehensive follow-up procedures were implemented to minimize loss to follow-up and ensure the completeness of data collection. Participants were contacted through various channels, including email, phone calls, and in-person reminders, to encourage their participation in the follow-up assessments.

### 2.4 Data collection

**Figure 1:**
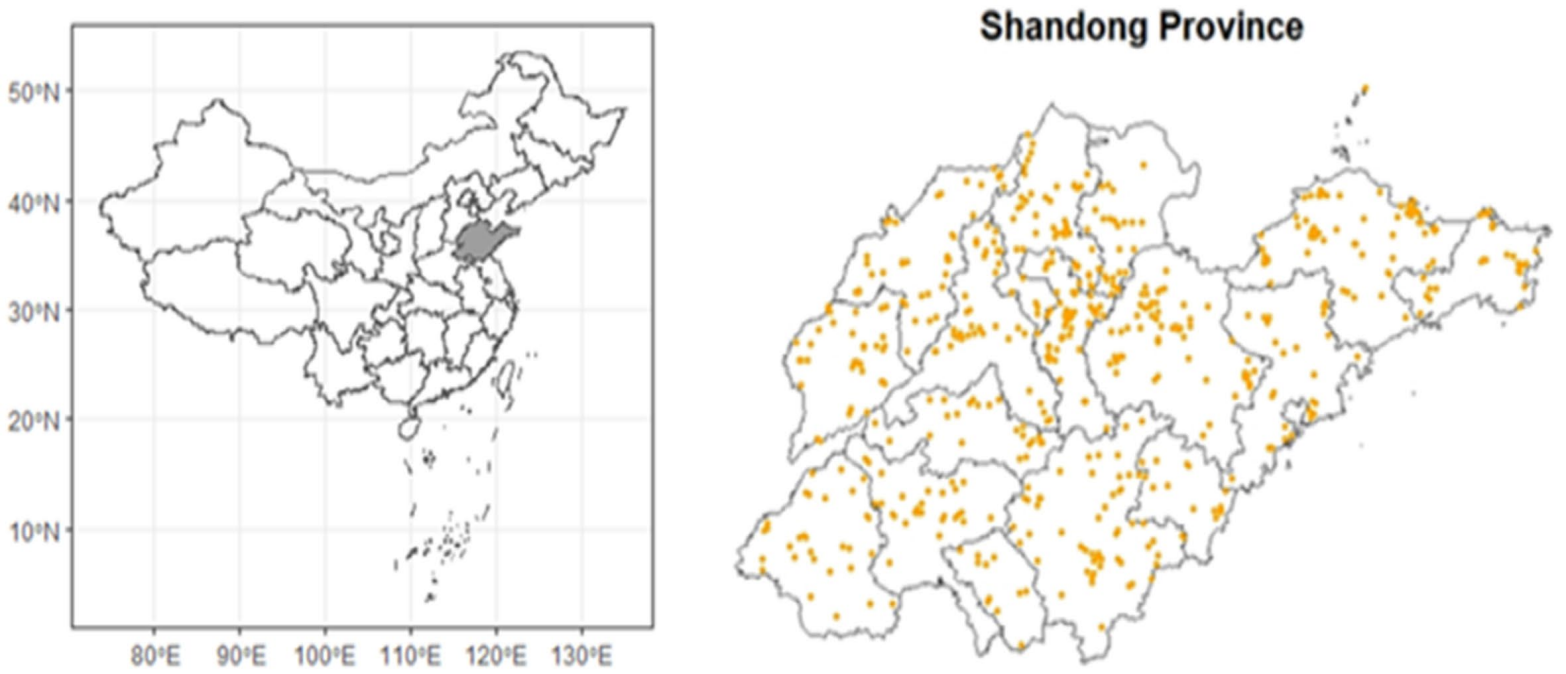
The geographical distribution of participants.

The study focused on undergraduate students enrolled in a specific university in Yantai City, Shandong Province, China, during the year 2019. Data collection occurred at two key time points: baseline in September 2019, and follow-up in September 2021. The geographical distribution of participants is illustrated in Figure 1.

To be eligible for inclusion in the study, participants had to meet the following criteria: (1) their home addresses were officially registered before university enrollment, (2) they were aged over 18 years, all having completed at least their sophomore year, and (3) demonstrated voluntary participation in the study along with the capability to complete a one-year follow-up.

### 2.5 Survey tools and indicators

Participants’ general demographic characteristics, encompassing age, gender, educational level, active and passive smoking status, alcohol consumption, physical activity, economic income, and seafood consumption, were gathered through detailed questionnaires.

For the assessment of sleep quality, the Pittsburgh Sleep Quality Index (PSQI) was utilized. It includes seven sleep dimensions and a total of 19 entries. The overall score ranges from 0 to 21, with higher scores indicating poorer sleep quality. A score of 7 was considered the reference cut-off value for identifying adult sleep problems in China.

Depression symptoms were evaluated using the Patient Health Questionnaire-9 (PHQ-9), which comprises nine entries with a total score of 27 points. A reference cut-off value of 4 points was established for identifying depression problems.

Anxiety levels were assessed using the Generalized Anxiety Disorder-7 (GAD-7), consisting of seven entries and a total score of 21 points. A reference cut-off value of 4 points was set for identifying anxiety problems.

### 2.6 Statistical analysis

Statistical analyses were conducted using R software (version 4.0.3) and SPSS 23.0. The significance level for two-tailed tests was set at *p* < 0.05. Categorical data were presented as frequencies and percentages, while continuous data were expressed as means and standard deviations.

### 2.7 Ethical considerations

This research received ethical approval from the BinZhou Medical University Ethics Committee (Code: 2019.45). Before their involvement, all participants provided written informed consent, emphasizing the voluntary nature of their participation. To safeguard participant privacy, the survey was conducted anonymously, and stringent measures were implemented to ensure the confidentiality of personal data through coding. The integrity of information was meticulously preserved at every stage of data collection and analysis.

## 3 Results

### 3.1 General information

A total of 692 participants were enrolled in this study, comprising 440 male and 252 female students. Notably, there were observed changes in certain lifestyle factors between 2019 and 2021. Specifically, students’ fruit and fish intake showed an increase in 2021 compared to 2019. However, water intake and physical activity among students decreased during the same period. Interestingly, alcohol consumption exhibited a decrease in 2021 (n = 9) compared to 2019 (n = 26). Conversely, both smoking and passive smoking showed an increase in 2021 compared to 2019. A summary of the participants’ characteristics is presented in Table 1.

**Table 1:** Baseline characteristics of study participants.

Characteristic		2019	2021
Gender	Male	440	440
	Female	252	252
Height (cm)		168 ± 8.58	168 ± 8.87
Weight (kg)		63.57 ± 15.57	63.75 ± 16.80
Age		18.37 ± 0.63	20.41 ± 0.83
BMI		22.32 ± 4.63	22.28 ± 4.53
Smoking	YES	7	15
	NO	685	677
Passive smoking	YES	607	658**
	NO	85	34
Alcohol consumption	YES	26	19
	NO	666	673
Physical activity (%)	YES	517	378**
	NO	175	314
Daily water intake	≤250 mL	35	42**
	250 ~ 750 mL	274	357
	750 ~ 1,500 mL	187	163
	>1,500 mL	196	130
Fruit Consumption (%)	≥Once a week	570	609**
	<Once a week	122	83
Fish Consumption (%)	≥Once a week	605	622
	<Once a week	87	70

^**^p < 0.01, ^*^p < 0.05.

### 3.2 Results of one-way analysis of college students’ sleep, anxiety, and depression levels

The results of data analysis revealed statistically significant differences in the levels of sleep, anxiety, and depression among college students when grouped by gender (only male students, 2.64 ± 2.54**), exercise (2.14 ± 2.48**,2.48 ± 4.03**,1.75 ± 3.40**), passive smoking (4.91 ± 6.10**, 4.56 ± 5.14**) and fruit intake (2.30 ± 2.51*, 2.76 ± 4.45**, 1.99 ± 3.74*) (*p* < 0.05). Additionally, significant differences in sleep (2.32 ± 2.54*) were observed among college students when categorized by seafood intake (*p* < 0.05), as detailed in Table 2.

**Table 2:** One-way analysis of college students’ sleep, anxiety and depression levels (2021).

Characteristic		PSQI	PHQ-9	GAD-7
Gender	Male	2.64 ± 2.54^**^	2.85 ± 4.14	1.95 ± 3.44
	Female	1.98 ± 2.72	3.08 ± 5.03	2.43 ± 4.35
Smoking	YES	1.60 ± 2.56	4.20 ± 7.13	3.27 ± 6.09
	NO	2.42 ± 2.62	2.90 ± 4.41	2.10 ± 3.74
Passive smoking	YES	1.76 ± 2.20	4.91 ± 6.10^**^	4.56 ± 5.14^**^
	NO	2.44 ± 2.63	2.83 ± 4.37	2.00 ± 3.68
Alcohol consumption	YES	1.74 ± 2.31	2.00 ± 3.09	1.47 ± 2.50
	NO	2.42 ± 2.63	2.96 ± 4.52	2.15 ± 3.83
Physical activity (%) Daily water intake	YES	2.14 ± 2.48^**^	2.48 ± 4.03^**^	1.75 ± 3.40^**^
	NO	2.72 ± 2.75	3.48 ± 4.93	2.59 ± 4.19
	≤250 mL	2.79 ± 3.47	3.52 ± 6.09	2.45 ± 5.18
	250 ~ 750 mL	2.17 ± 2.40	2.82 ± 4.59	2.06 ± 3.85
	750 ~ 1,500 mL	2.71 ± 2.76	2.68 ± 3.71	2.02 ± 3.33
	>1,500 mL	2.54 ± 2.69	3.35 ± 4.49	2.35 ± 3.74
Fruit Consumption	≥Once a week	2.30 ± 2.51^*^	2.76 ± 4.45^**^	1.99 ± 3.74^*^
	<Once a week	3.14 ± 3.27	4.17 ± 4.62	3.13 ± 4.11
Fish Consumption	<Once a week	2.32 ± 2.54^*^	2.82 ± 4.27	2.04 ± 3.63
	≥Once a week	3.13 ± 3.20	3.91 ± 6.01	2.94 ± 5.03

^**^p < 0.01, ^*^p < 0.05.

### 3.3 Analysis of the results of changes in the sleep condition, anxiety, and depression levels of college students

The results of the data analysis indicate a statistically significant difference in the sleep quality of college students between the beginning of enrollment (3.30 ± 2.08), and the follow-up (2.40 ± 2.62) period (*p* < 0.05). However, no significant changes were observed in the anxiety and depression levels during this timeframe, as detailed in Table 3.

**Table 3:** The changes in sleep condition, anxiety, and depression levels of college students.

Characteristic	2019	2021	*t/z*	*p*-value
PSQI	3.30 ± 2.08	2.40 ± 2.62	−9.10	0.00**
PHQ-9	1.78 ± 3.07	2.13 ± 3.80	−0.33	0.74
GAD-7	2.76 ± 3.61	2.93 ± 4.48	−1.82	0.07

^**^p < 0.01, *p < 0.05.

### 3.4 The influence of college students sleep status on depression level

In the regression analysis, variables exhibiting statistically significant differences in both one-way analysis and correlation analysis—namely PSQI (0.55, CI0.43, 0.67), passive smoking (2.46, CI1.00, 3.91), exercise (0.68, CI0.04, 1.31), and fruit intake (0.87, CI-0.10, 1.84) were employed as independent variables. The dependent variables included anxiety and depression levels. The results of the regression analysis identified that PSQI, passive smoking, and exercise entered into the regression equation as influencing factors on the anxiety levels of college students (*p* < 0.01). The R2 value was calculated to be 0.13, collectively explaining 13% of the variance, as illustrated in Table 4.

**Table 4:** The influence of college students sleep status on depression level (2021).

Characteristic	Beta	B (95%CL)	*p*-value
PSQI	0.32	0.55 (0.43, 0.67)	0.00^**^
Passive smoking	0.12	2.46 (1.00, 3.91)	0.00^**^
Physical activity (%)	0.08	0.68 (0.04, 1.31)	0.03^*^
Fruit Consumption	0.06	0.87(−0.10, 1.84)	0.08

^**^p < 0.01, ^*^p < 0.05.

### 3.5 The influence of college students’ sleep status on anxiety levels

The regression analysis results reveal that PSQI (*p* = 0.00**), passive smoking (0.00**), and exercise (0.02*) are significant factors influencing the depression levels of college students (p < 0.01). The regression equation includes these variables, and the R2 value is calculated to be 0.12, collectively explaining 12% of the variance. The detailed findings are presented in Table 5.

**Table 5:** The Influence of college students sleep status on anxiety levels (2021).

Characteristic	Beta	B (95%CL)	*p*-value
PSQI	0.28	0.39 (0.28, 0.49)	0.00^**^
Passive smoking	0.16	2.82 (1.58, 4.07)	0.00^**^
Physical activity (%)	0.08	0.61 (0.07, 1.16)	0.02^*^
Fruit Consumption	0.06	0.58(−0.25, 1.42)	0.08

^**^p < 0.01, ^*^p < 0.05.

## 4 Discussion

### 4.1 The results of college students’ sleep condition, anxiety and depression level

The study findings underscore several influential factors on college students’ sleep status, anxiety, and depression levels, with notable associations observed. Women exhibited significantly lower sleep quality than men. Additionally, regular exercise demonstrated a positive impact on sleep quality, aligning with similar observations in studies conducted by Castelli Lucia and Qi Feng (Feng et al., 2014; Castelli et al., 2023). Dietary habits also emerged as crucial factors in this study, with higher sleep quality reported among individuals consuming fruits and seafood at least once a week. This is consistent with the results of surveys by Doaky and Hepsomali Piril, emphasizing the positive effects of increased intake of vegetables, fruits, and fish on promoting sleep health (Hepsomali and Groeger, 2021; Doak et al., 2023). Regarding anxiety and depression levels, the study identified passive smoking, exercise, and fruit intake as influential factors. College students exposed to passive smoking exhibited significantly higher scores for anxiety and depression, while those engaging in regular exercise and consuming fruits demonstrated lower scores for anxiety and depression levels. These findings align with the results of Dominika Głąbska’s systematic review on vegetable and fruit intake and mental health in adults, as well as Meg Fluharty and Matthew Pearce’s systematic review on the correlation between smoking, exercise, and anxiety and depression (Głąbska et al., 2020). The establishment of positive living habits, including regular exercise and a diet rich in fruits and seafood, has demonstrated a consistently positive impact on college students’ sleep quality, anxiety, and depression levels. These results align with existing research both domestically and internationally, emphasizing the importance of cultivating healthy lifestyle practices for the well-being of college students (Fluharty et al., 2017; Pearce et al., 2022).

### 4.2 Analysis of the differences in the changes in college students’ sleep status, anxiety, and depression levels

This study reveals a noteworthy difference in the sleep condition of college students between the early stage of enrollment and the follow-up period. Specifically, the sleep quality score significantly decreased during the follow-up stage, indicating an improvement in sleep quality. However, anxiety and depression scores did not exhibit significant changes compared to the earlier phase. Considering the distinctive features of the domestic education system and the variations in the teaching environment between high school and university, it’s essential to contextualize these findings. A domestic scholar (Liu et al., 2020) investigating the sleep status of high school students reported a high prevalence of sleep abnormalities, reaching 17.2%, with the domestic norm standing at 45.6%. In contrast, Xiaoyan Wu (Wu et al., 2015) in a study on the sleep quality of college students in China, found a lower percentage of sleep abnormalities at 9.8%, notably lower than the levels observed among high school students. This discrepancy is attributed to the reduced academic burden and improved lifestyle rhythm experienced by college students, positively influencing sleep quality. Furthermore, the study identified significant differences in passive smoking, exercise, fruit intake, and daily water intake among college students during the follow-up stage compared to the early enrollment period. Notably, there was a substantial increase in the number of individuals engaging in regular exercise and consuming fruits during the follow-up stage. Considering the recognized influence of regular exercise and fruit intake on sleep quality, these changes may be correlated with the observed improvements in the sleep quality of college students. It’s important to note that anxiety and depression scores did not demonstrate significant changes in this study. While the literature search did not yield specific analyses comparing anxiety and depression status between high school and university stages, further research is warranted to provide a more comprehensive understanding of these aspects during the transition from high school to university.

### 4.3 Analysis of the role of college students’ sleep condition on anxiety and depression levels

Students and their levels of anxiety and depression. Notably, poorer sleep quality is associated with higher levels of anxiety and depression, aligning with similar observations in both domestic and international research. For instance, in a Meta-analysis conducted by foreign scholar Alexander J. Scott, the improvement of sleep quality demonstrated a moderately significant effect on mental health, specifically reducing anxiety and depression (Scott et al., 2021). The results of regression equations in our study emphasize the impact of various factors on anxiety and depression levels during the follow-up period. PSQI scores, passive smoking, and exercise collectively accounted for a 12% influence effect size, with sleep contributing to 10% of the total. Contrastingly, at the beginning of the school year, sleep exerted a more substantial influence on effect size ranging from 16 to 26% on anxiety and depression levels. This underscores the dynamic nature of the relationship between sleep quality and mental health over time.

Existing domestic and international studies have consistently affirmed the correlation between sleep quality and anxiety and depression levels. Notably, effective improvements in sleep quality have been shown to positively impact the prevention of anxiety and depression. Given that college students are navigating a phase of rapid self-conscious development but may lack proper guidance in personal values, they are particularly susceptible to the influences of environmental changes, leading to the emergence of psychological challenges. Consequently, a comprehensive understanding and correct interventions are crucial for promoting the mental well-being of college students (Palagini et al., 2022).

Current study has many strengthen points such as: The study utilized a prospective design, allowing for the longitudinal assessment of sleep status, anxiety, and depression levels among college students. Secondly, with a total of 692 participants, the study had a sizable sample size, enhancing the reliability and generalizability of the findings. 3rd, The use of standardized tools, including the Pittsburgh Sleep Quality Index (PSQI), Patient Health Questionnaire-9 (PHQ-9), and Generalized Anxiety Disorder-7 (GAD-7), ensured comprehensive evaluation of sleep conditions, anxiety, and depression levels.

This study also has limitation such as: The study was conducted at a single university in Shandong Province, which may limit the generalizability of the findings to other populations or regions. Another limitation of this study is related to the timing of data collection, which coincided with the SARS-CoV-2 pandemic. The unprecedented circumstances surrounding the pandemic, including widespread disruptions to daily life, healthcare services, and socioeconomic activities, may have influenced participants’ behaviors, health outcomes, and access to healthcare resources. These external factors could have introduced confounding variables and affected the generalizability of the study findings. Additionally, restrictions imposed to mitigate the spread of the virus, such as lockdowns and social distancing measures, might have impacted the availability and reliability of data, as well as the ability to conduct follow-up assessments. Therefore, while every effort was made to ensure data quality and accuracy, the pandemic context should be acknowledged as a potential limitation in interpreting the study results.

## 5 Conclusion

In this study, we focused on college students in the early stages of enrollment, conducting a thorough 2-year follow-up observation to gain valuable insights into the changes in their sleep status, anxiety, and depression levels. Our findings highlighted gender, passive smoking, exercise, fruit intake, and seafood consumption as influential factors in these aspects of college students’ well-being. Notably, during the follow-up period, we observed a significant improvement in the sleep quality of college students compared to the early stages of enrollment, offering valuable guidance for clinical practice.

While this study presents a prospective follow-up survey, it is essential to acknowledge its limitation of having completed only one follow-up assessment, which may constrain its capacity to guide long-term changes effectively. To enhance the accuracy of our results and provide more comprehensive guidance, we recommend future research endeavors to incorporate multiple follow-up surveys, spanning from the early stages of enrollment to graduation. This approach would offer a more nuanced understanding of the dynamic nature of sleep, anxiety, and depression levels among college students over an extended period.

## Data availability statement

The original contributions presented in the study are included in the article/supplementary material, further inquiries can be directed to the corresponding author.

## Ethics statement

The studies involving humans were approved by the BinZhou Medical University Ethics Committee (code: 2019.45). The studies were conducted in accordance with the local legislation and institutional requirements. The participants provided their written informed consent to participate in this study.

## Author contributions

CZ: Data curation, Formal analysis, Investigation, Writing – review & editing. LZ: Investigation, Methodology, Validation, Writing – review & editing. TD: Investigation, Software, Visualization, Writing – original draft. JZ: Investigation, Methodology, Writing – original draft. CG: Methodology, Software, Writing – review & editing. FZ: Conceptualization, Supervision, Writing – original draft.
